# Evaluation of advanced platelet-rich fibrin (PRF) as a bio-carrier for ampicillin/sulbactam

**DOI:** 10.1007/s00784-022-04663-y

**Published:** 2022-08-09

**Authors:** Anton Straub, Andreas Vollmer, Thiên-Trí Lâm, Roman C. Brands, Maximilian Stapf, Oliver Scherf-Clavel, Max Bittrich, Andreas Fuchs, Alexander C. Kübler, Stefan Hartmann

**Affiliations:** 1 Department of Oral and Maxillofacial Plastic Surgery of the University Hospital Würzburg, Pleicherwall 2, 97070 Würzburg, Germany; 2 Institute for Hygiene and Microbiology of the University of Würzburg, Josef-Schneider-Street 2/E1, 97080 Würzburg, Germany; 3 Institute of Pharmacy and Food Chemistry of the University of Würzburg, Am Hubland, 97074 Würzburg, Germany; 4 Department of Internal Medicine II, University Hospital Würzburg, Josef-Schneider-Street 2, 97080 Würzburg, Germany

**Keywords:** Osteonecrosis of the jaw, Osteoradionecrosis, Antiresorptive drug-related osteonecrosis of the jaw, ARONJ, Oral microbiome, Agar diffusion test

## Abstract

**Objectives:**

Mechanisms of wound healing are often impaired in patients with osteonecrosis of the jaw (ONJ). According to the guidelines for the treatment of this disease, early surgical intervention is indicated. However, surgery often faces complications such as wound healing disorders. The application of platelet-rich fibrin (PRF) after necrosectomy between bone and mucosa may constitute a promising approach to improve surgical results. An aspect that was not investigated until now is that PRF acts as a “bio-carrier” for antibiotics previously applied intravenously.

**Materials and methods:**

We investigated the antimicrobial properties of PRF in 24 patients presenting ONJ undergoing systemic antibiosis with ampicillin/sulbactam. We measured the concentration of ampicillin/sulbactam in plasma and PRF and performed agar diffusion tests. Ampicillin/sulbactam was applied intravenously to the patient 10 minutes for blood sampling for PRF. No further incorporation of patients’ blood or PRF product with antibiotic drugs was obtained. Four healthy patients served as controls.

**Results:**

Our results revealed that PRF is highly enriched with ampicillin/sulbactam that is released to the environment. The antibiotic concentration in PRF was comparable to the plasma concentration of ampicillin/sulbactam. The inhibition zone (IZ) of PRF was comparable to the standard ampicillin/sulbactam discs used in sensitivity testing.

**Conclusions:**

The results of our study demonstrated that PRF is a reliable bio-carrier for systemic applied antibiotics and exhibits a large antimicrobial effect.

**Clinical relevance:**

We describe a clinically useful feature of PRF as a bio-carrier for antibiotics. Especially when applied to poorly perfused tissues and bone such as in ONJ, the local release of antibiotics can reduce wound healing disorders like infections.

## Introduction

Wound healing is a complex process that includes many different mechanisms occurring in parallel at the cellular level to regenerate or repair the affected tissues. This complex is particularly prone to disruption in areas suffering from poor circulation. In patients with osteonecrosis of the jaw (ONJ), for example, this may occur following radiation to the head and neck area or antiresorptive drug intake. These drugs are usually bisphosphonates (BP) or denosumab, which are used in the treatment of diseases that alter bone metabolism, such as bone metastases, osteoporosis, multiple myeloma, or fibrous dysplasia [1, 2]. The inhibitory effect of BP on mature osteoclasts and the inhibitory effect of denosumab on RANK ligand can lead to the complication of antiresorptive drug-related osteonecrosis of the jaw (ARONJ) [3]. This disorder is characterized by the exposure of necrotic bone for at least eight weeks and the progressive involvement of the surrounding bone structures without prior radiation treatment to the head [3]. Owing to the lack of protection afforded by the soft tissue cover, the bone is exposed to the microbial flora of the oral cavity. This can lead to the development of infections and bacterial colonies, even if systemic antibiotic treatment has been administered [4]. Early surgical intervention can reduce bone and tooth loss, and the standard procedure adopted is normally necrosectomy and mucosal closure [5]. However, this procedure often leads to complications such as wound healing disorders or (re-)exposed bone [6]. These statements can also be applied to patients with ONJ following radiation therapy (osteoradionecrosis, ORN), even if the reason of osteonecrosis is different.

**Fig. 1:**
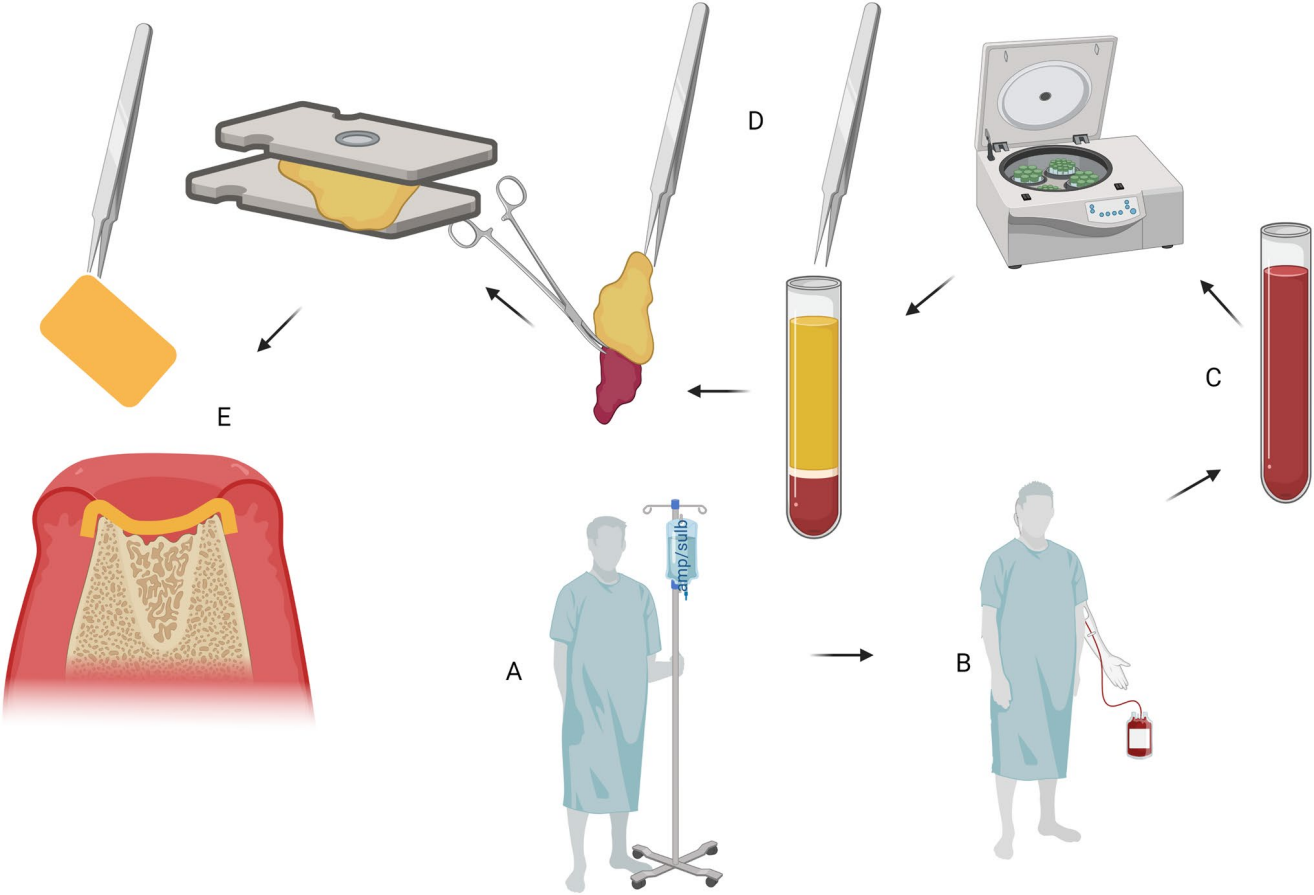
Flow chart of the manufacturing process for PRF. A: Antibiotics (ampicillin/sulbactam 3g, every eight hours) were intravenously applied to the patient at least three times. B: 10 minutes after an additional injection of ampicillin/sulbactam blood sample for PRF was performed. C: Blood was centrifuged (A-PRF+ protocol, Duo Quattro centrifuge). D: Solid PRF membranes were fabricated in a usual manner. E: The firm membranes were then placed on the bone. This could be beneficial because of the release of growth factors and the coverage of sharp bone edges. All illustrations were produced independently by the authors, and require the citation term “created with BioRender.com”

In addition to the standard surgical procedure, platelet-rich fibrin (PRF) may be used to improve the surgical outcome by supporting the regeneration of bony and soft tissue structures, which is particularly important in regions of poor circulation [7–9]. The use of PRF has become increasingly influential in the surgical disciplines of dentistry and maxillofacial surgery [10, 11]. PRF is obtained from whole blood through centrifugation. In this process, the macro components, such as red blood cells, are separated from the plasma components. This produces a suspension enriched with white blood cells and growth factors. The fibrin matrix of PRF allows the growth factors to be released over several days or even weeks and can give the product, depending on centrifugation force applied and protocol, a firm structure [10, 12]. For application after necrosectomy, it is possible to form membranes, which may be applied to the bone (Fig. 1). Besides the release of growth factors, this PRF layer acts as an additional barrier and coverage of sharp bone edges, which improves mucosal closure. Various authors have already reported promising results with PRF in ARONJ [13, 14].

Even though the full effect mechanism is not yet fully understood, PRF has been demonstrated to possess antimicrobial features without additional work-up or preparation [15–17]. Studies have demonstrated that PRF has a bactericidal and growth-inhibiting effect against both biofilm-forming and periodontal microorganisms. It was also concluded that PRF may be used to prevent postoperative wound infections [18, 19]. Furthermore, an attempt was made to extend this antibacterial feature by adding antibiotics to the suspension after centrifugation. The idea here was to employ PRF as an antibacterial carrier to attain the retarded release of the active ingredient at the site of delivery [20, 21].

To avoid this additional step, we investigated whether antibiotics can be detected within the PRF in potentially the same concentration as in patients’ blood after systemic application. And whether thus achieve the targeted application of the active substance locally at the required site. This is especially important in poorly perfused tissues as it can be found in patients with ONJ, regardless of their aetiology (radiation or medication-related). ONJ patients are particularly suitable for this study because they mostly undergo antibiosis regardless of further treatment. To the best of our knowledge, this is the first study to investigate whether PRF enhances systemically applied antibiotics and whether the local antimicrobial effect thus unleashed is sufficient as treatment.

## Methods

**Fig. 2:**
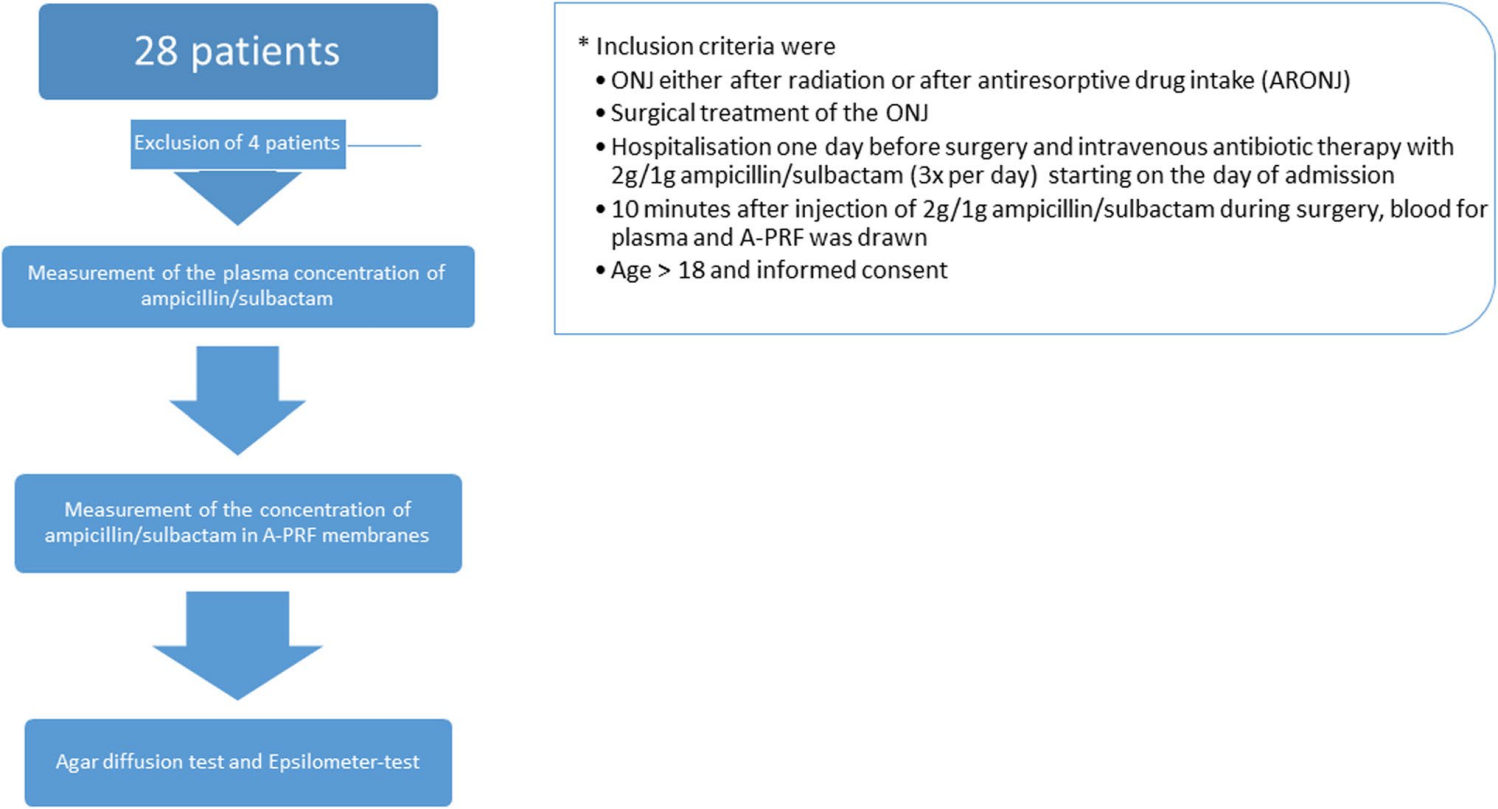
Flow chart of the study protocol. Twenty-eight patients fulfilled the inclusion criteria and were enrolled in this study. Four patients had to be excluded because sample collection was insufficient (e.g. wrong storage temperature, too long storage). In three cases, no sufficient PRF could be produced. Thus, from 24 patients, 21 PRF membranes were used for further examination. Furthermore, four PRF membranes were necessary to establish the pharmaceutical approach and methods to measure the concentration of ampicillin and sulbactam in the PRF membranes. Thus, 17 PRF membranes were analysed in total. PRF membranes from 10 patients could be processed for agar diffusion tests

We investigated the effects of PRF as a bio-carrier for ampicillin/sulbactam in a prospective trial comprising a total of 28 patients suffering from ONJ, who were treated in the University Hospital of Würzburg in the period from October 2020 to November 2021 (Fig. 2). Furthermore, four patients not suffering from ONJ and without antibiotic therapy served as control group.

We set a diagnosis of osteonecrosis of the jaw (ONJ), either after radiation (ORN) or after medication with antiresorptive agents (ARONJ), and the surgical treatment of this ONJ, as well as an age at onset of at least 18 years and the provision of written informed consent as inclusion criteria. Patients were excluded from participating if allergic to penicillin, if the cause of their ONJ was anything other than ORN or ARONJ, such as osteomyelitis, or when there was a failure to comply with protocols in the study after being included (e.g. too long or wrong blood/PRF storage, wrong blood sampling).

The Ethics Committee of the University of Würzburg approved all the protocols implemented in this study (IRB approval number: 143/20-me).

### Antibiotic therapy

Participants were admitted to hospital one day prior to surgical intervention and intravenous antibiotic therapy with ampicillin and sulbactam (Unacid® Pfizer Pharma GmbH, Berlin, Germany), 3g, every eight hours was started on the day of admission. During surgery, 3g Unacid® was again administered as perioperative prophylaxis. According, to our protocol, every participant received at least three doses of ampicillin/sulbactam 2/1g and one additional shot 10 minutes before plasma and PRF blood sampling.

## Plasma

Blood sampling for plasma and PRF was performed 10 minutes after intravenous injection of 2g/1g ampicillin/sulbactam intraoperatively. Blood for the plasma sample was collected after venepuncture in a 1.6ml EDTA tube (S-Monovette, Sarstedt, Sarstedt-Straße 1, 51588 Nümbrecht, Germany) and centrifuged (4900 rpm for 10 minutes and 4°C). Four aliquots of 100 μl were frozen at -80 C. The concentration of ampicillin and sulbactam was measured at the Institute of Pharmacy of the University of Würzburg (see “*Quantification of ampicillin/sulbactam plasma and PRF levels”).*

## Advanced platelet-rich fibrin plus (A-PRF+)

**Fig. 3:**
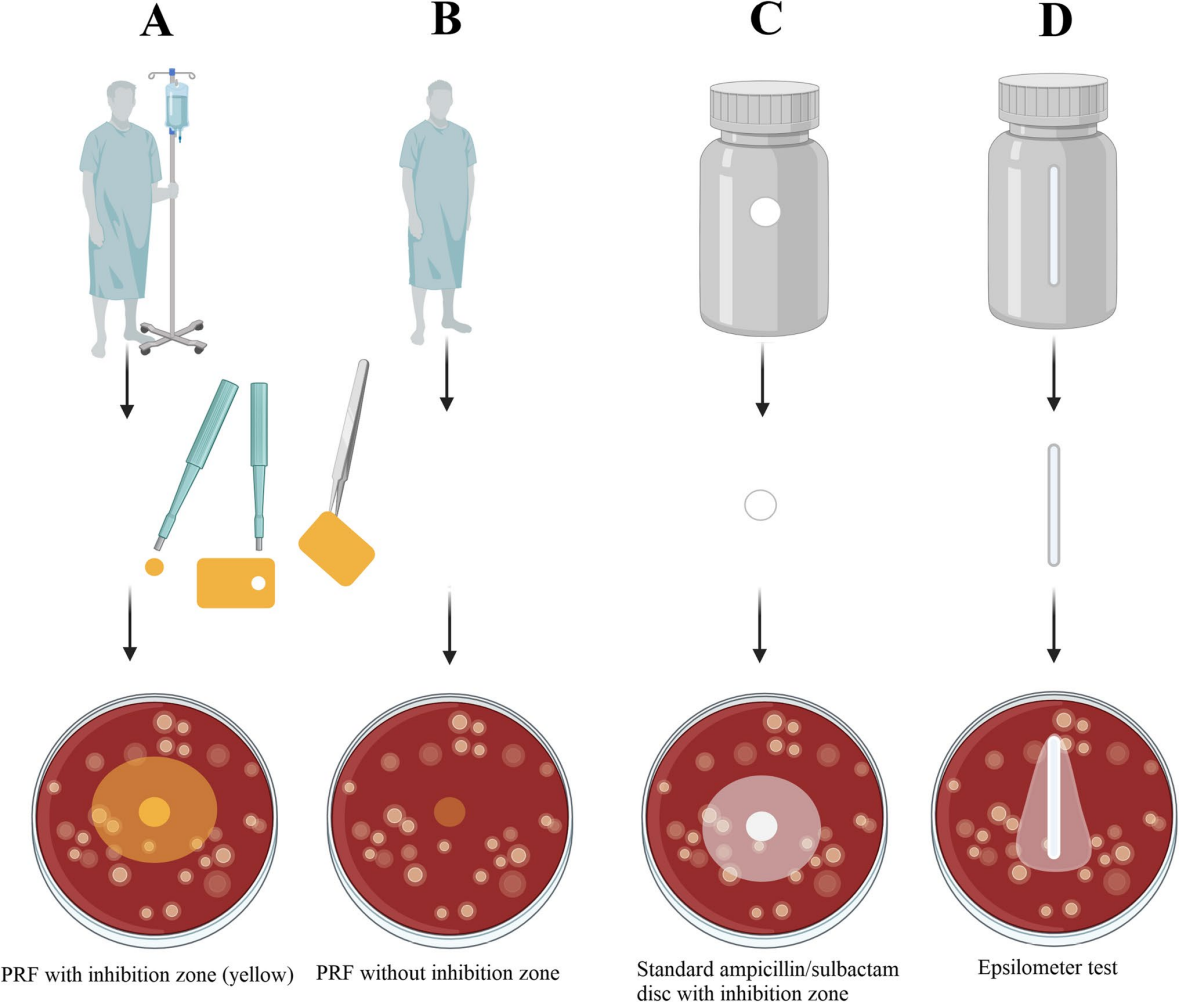
Flow chart for the analysis of the results with colony forming units. A: PRF obtained from a patient with intravenous antibiotic therapy (ampicillin/sulbactam). PRF of these patients showed an inhibition zone in agar diffusion tests. B: Control group with PRF from patients without previous antibiotic administration and therefore, without showing an inhibition zone C: standard ampicillin/sulbactam disc (20μg) showing a regular inhibition zone in agar diffusion test D: Epsilometer test to determine the minimum inhibitory concentration. All illustrations were produced independently by the authors, and require the citation term “created with BioRender.com”

Blood for the production of PRF was sampled together with the EDTA tube intraoperatively 10 minutes after the perioperative prophylaxis of 2g/1g ampicillin/sulbactam was injected to the patient (see above). For PRF blood sampling four to six sterile 10ml vacuum glass A-PRF tubes (Process for PRF, Nice, France) were used. A-PRF was obtained by a Duo Quattro centrifuge (Process for PRF, Nice, France) with A-PRF+ protocol (1300rpm for 8 minutes, RCF-max = 208g, 40° rotor angulation with a radius of 77mm at the clot and 110mm at the max). Blood clots were pressed and PRF membranes were fabricated in the usual manner (Fig. 1 and Fig. 3) [22].

Depending on the number of PRF tubes sampled (surgeon’s choice), four to six PRF membranes could be produced. One of these membranes was placed natively into an Eppendorf Tube® and frozen at -80°C. Plasma and PRF samples were sent to the Institute for Pharmacy and Food Chemistry afterwards. Specimens were stored at -20°C during transport (duration 1h). For the further process, see **“***Quantification of ampicillin/sulbactam plasma and PRF levels”*. From another PRF membrane, four 6mm discs were cut out with a biopsy punch (kai Europe GmbH, Solingen, Germany, Fig. 3). These PRF discs were also placed natively in an Eppendorf Tube® and were sent to the Institute of Microbiology of the University of Würzburg for the agar diffusion tests (see “*Agar diffusion test”*) directly after the manufacturing process (duration 30-60 minutes).

## Quantification of ampicillin/sulbactam levels in plasma and PRF

A specific liquid chromatography–tandem mass spectrometry (LC–MS/MS) method was developed and validated according to the European Medicines Agency guidelines on bioanalytical method validation [23].

Samples were monitored through electrospray ionization in the multiple-reaction-monitoring mode. Ampicillin was measured in the positive-ion mode and sulbactam in the negative-ion mode (MRM transitions used for quantification: m/z 350.0→106.0 for ampicillin; m/z 355.2→111.0 for the corresponding internal standard ampicillin-d5; m/z 231.9→63.8 for sulbactam; m/z 236.8→63.7 for internal standard sulbactam-d5). Protein precipitation was applied for the sample preparation of plasma as well as for the PRF matrix (acetonitrile for plasma and methanol 80% for PRF). In the case of PRF, samples were homogenized prior to further processing using an IKA ULTRA-TURRAX® disperser after an appropriate volume of phosphate-buffered saline (PBS) solution had been added. The lower limit of quantification (LLOQ) of the plasma method was 2μg/ml for both ampicillin and sulbactam. Regarding the PRF method, LLOQ values for ampicillin and sulbactam were 1μg/ml and 2μg/ml, respectively.

## Agar diffusion test

Agar diffusion tests were performed with *Haemophilus influenzae* ATCC 49766, *Staphylococcus aureus* ATCC 29213, *Streptococcus pneumoniae* ATCC 49619, and *Escherichia coli* ATCC 25922 according to the EUCAST disc agar diffusion methodology [24]. Bacterial suspensions were adjusted to a McFarland 0.5 turbidity standard using a DensiCHEKTM Plus instrument (bioMérieux, Nürtingen, Germany) in 0.85% NaCl w/v in water. The inocula of *S. aureus* and *E. coli* were plated on unsupplemented Mueller–Hinton E agar (MH-E, bioMérieux, Nürtingen, Germany), *Pneumococcus* and *Haemophilus influenzae* were plated on Mueller–Hinton agar with 5% defibrinated horse blood and 20mg/l β-NAD (MH-F, BD, Heidelberg, Germany). The inoculum was spread evenly over the entire surface of the agar plate using a cotton swab. A 6mm PRF disc, was placed on each inoculated plate. As technical controls, a disc agar diffusion test was performed in parallel using an antimicrobial susceptibility test disc (Thermo Scientific Oxoid, Langenselbold, Germany) loaded with 20μg ampicillin/sulbactam, as well as a gradient agar diffusion test (Epsilometer test) using a test strip (Liofilchem, Roseto degli Abruzzi, Italy) loaded with an ampicillin gradient ranging from 0.016 to 256mg/l, and a fixed sulbactam load of 4mg/l (Fig. 3). Agar plates were incubated at 35° for 20 hours with ambient air (*Staphylococcus aureus*, *Escherichia coli*) or 5% CO_2_ atmosphere. Upon incubation, the respective diameters of the inhibition zones (IZ) were measured in millimetres and photographs were taken for documentation purposes (Fig. 3).

As a negative control, PRF from four healthy patients without antibiotic therapy was also processed for the agar diffusion tests as described above.

## Statistics

Descriptive statistical analyses were performed with GraphPad Prism, version 9 (GraphPad Software, San Diego, USA).

## Results

### Descriptive statistics

28 patients were initially enrolled in this study. We had to exclude four patients during the course of the trial, because of study protocol violations. The mean age was 69 with more males (53.6%) included. All participants suffered from osteonecrosis of the jaw, either after radiation (ORN) or following the intake of antiresorptive drugs (ARONJ) (Table 1). Furthermore, four patients not suffering from ONJ and without antibiotic therapy were included as controls.

**Table 1:** Descriptive statistics

	Participants
N (total)	24*
m/f	14/10
Mean age (in years)	69
ORN	7
ARONJ	17

N: number of participants, m: male, f: female, ORN: osteoradionecrosis, ARONJ: antiresorptive agent-related osteonecrosis of the jaw

*Four patients had to be excluded because the study protocol was not followed correctly (see *Methods*), thus 24 patients were enrolled in total.

## Ampicillin and sulbactam measurement in plasma and PRF

**Fig. 4:**
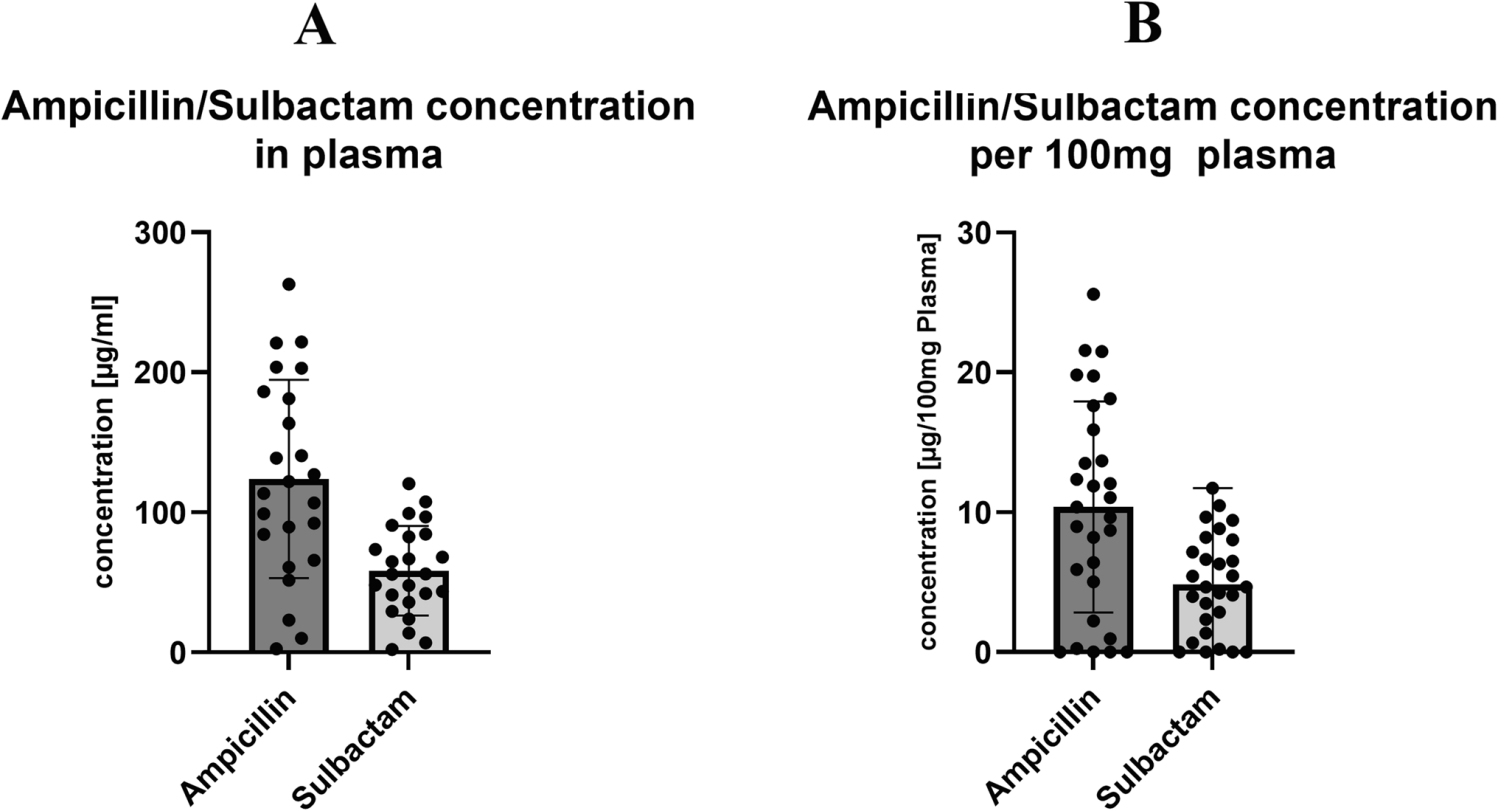
Plasma ampicillin and sulbactam concentration. A: The average concentration in plasma was 123.82μg/ml ampicillin (SD±70.79) and 58.45μg/ml sulbactam (SD±32.02). B: For better comparability with PRF the plasma concentration was converted to 100mg plasma. The adjusted concentration on 100mg plasma depicted was thus, on average, 12.05μg/100mg plasma for ampicillin and 5.67μg/100mg plasma for sulbactam assuming a plasma density of approximately 1028g/l

Measurement of ampicillin and sulbactam in the plasma of 24 patients revealed an average concentration of 123.82μg/ml ampicillin (SD±70.79) and 58.45μg/ml sulbactam (SD±32.02) (Table 2 and Fig. 4).

**Table 2:** Concentration of ampicillin and sulbactam in plasma

	Ampicillin	Sulbactam
N	24	24
Concentration (μg/ml)*	123.82	58.45
Concentration (μg/100mg Plasma**)	12.05	5.67
SD*	±70.79	±32.02
95% CI*	153.7 – 93.93	71.98 – 44.93

N: Number of patients. SD: Standard deviation. 95% CI: 95% confidence interval

*SD and 95% CI refer to the concentration in μg/ml

**To ease comparison with the concentration in PRF, ampicillin and sulbactam concentration was calculated in μg/100mg plasma (density 1028g/l) [25]

**Fig. 5:**
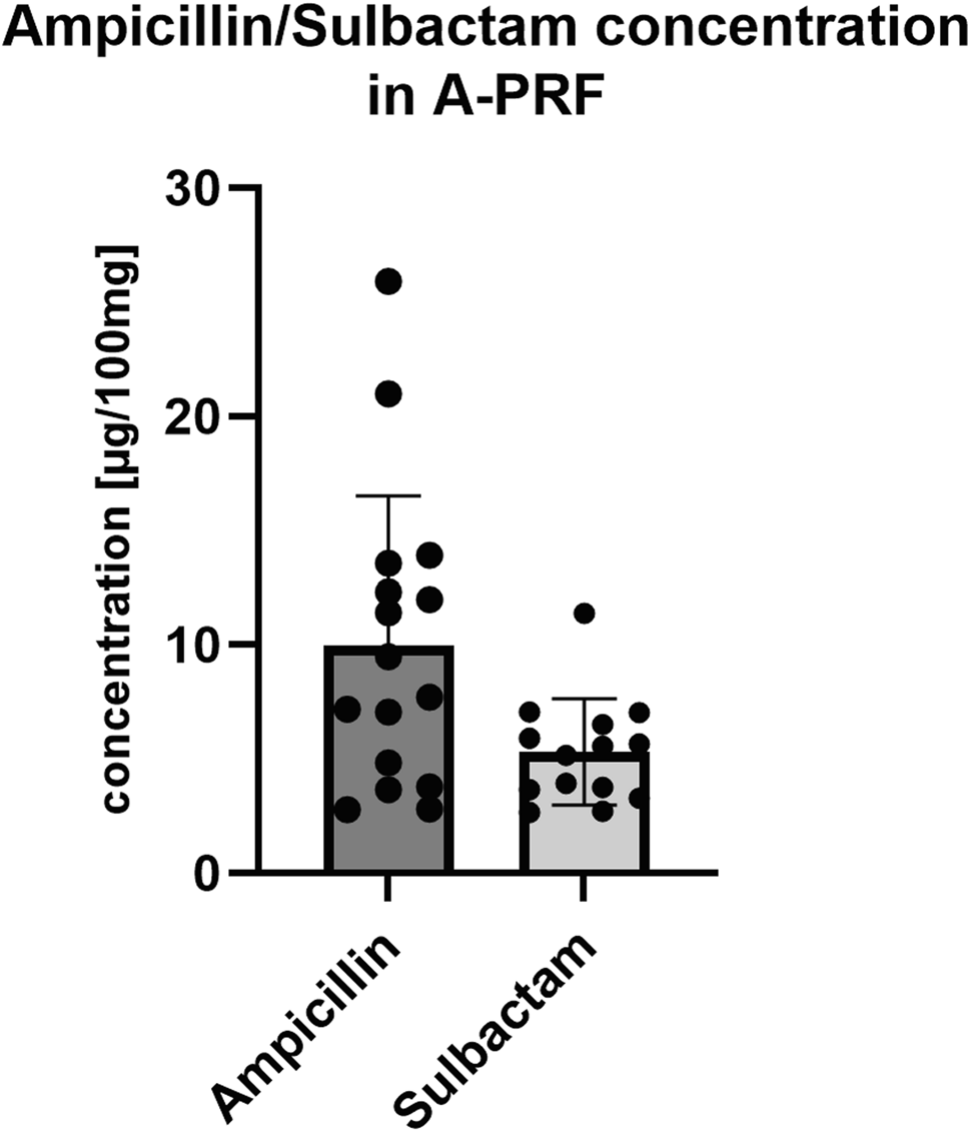
Ampicillin and sulbactam concentration in PRF per 100mg. The concentration of ampicillin and sulbactam in PRF was defined in μg/100mg, since the average PRF membrane weighed 100mg. Measurement revealed an average concentration of 13.52μg/100mg ampicillin (SD±15.96) and 5.31μg/100mg sulbactam (SD±2.32)

The concentration of ampicillin and sulbactam in PRF was defined in μg/100mg, since the average PRF membrane weighed 100mg. The measurement of 17, respectively, 14 patients revealed an average concentration of 13.52μg/100mg ampicillin (SD±15.96) and 5.31μg/100mg sulbactam (SD±2.32) (Table 3 and Fig. 5). The adjusted plasma ampicillin/sulbactam concentration was thus, on average, 12.05μg/100mg plasma for ampicillin and 5.67μg/100mg plasma for sulbactam assuming a plasma density of approximately 1028g/l (Table 2 and Fig. 4) [25].

**Table 3:** Concentration of ampicillin and sulbactam in PRF

	Ampicillin	Sulbactam
N	17	14
Concentration (μg/100mg PRF)*	13.52	5.31
SD	±15.96	±2.32
95% CI	21.72 – 5.31	6.65 – 3.97

N: Number of patients. SD: Standard deviation. 95% CI: 95% confidence interval

*Concentration of ampicillin and sulbactam was given in μg/100mg since the PRF membranes had an average mass of 100mg.

## Agar diffusion tests with monocultures of *Haemophilus influenzae*, *Streptococcus pneumoniae*, *Staphylococcus aureus*, and *Escherichia coli*

The bacteria tested displayed IZs and minimal inhibitory concentrations for ampicillin/sulbactam within the expected ranges in all tests. The average diameter of the IZ of the 6mm PRF disc was 23.22mm (SD±3.49), 19.56mm (SD±2.54), and 28.44mm (SD±3.94) for *Haemophilus influenzae*, *Streptococcus pneumoniae*, and *Staphylococcus aureus*, respectively (Table 4). An effect (IZ) was observed in all patients (10/10).

**Table 4:** Average diameter of the inhibition zone in the agar diffusion tests with PRF

	*Haemophilus influenzae*	*Staphylococcus aureus*	*Streptococcus pneumoniae*	*Escherichia coli*
N	9	9	9	10
inhibition zones (mm)	23.22	19.56	28.44	2.7
SD	±3.49	±2.54	±3.94	±4.37
95% CI	20.38-26.07	17.48-21.63	25.23-31.66	0-5.83
E-test (±SD) (mg/l)	0.19 (±0.02)	0.18 (±0.03)	0.07 (±0.01)	2.78 (±0.33)

N: Number of patients. SD: Standard deviation. 95% CI: 95% confidence interval. E-test: Epsilometer test

**Fig. 6:**
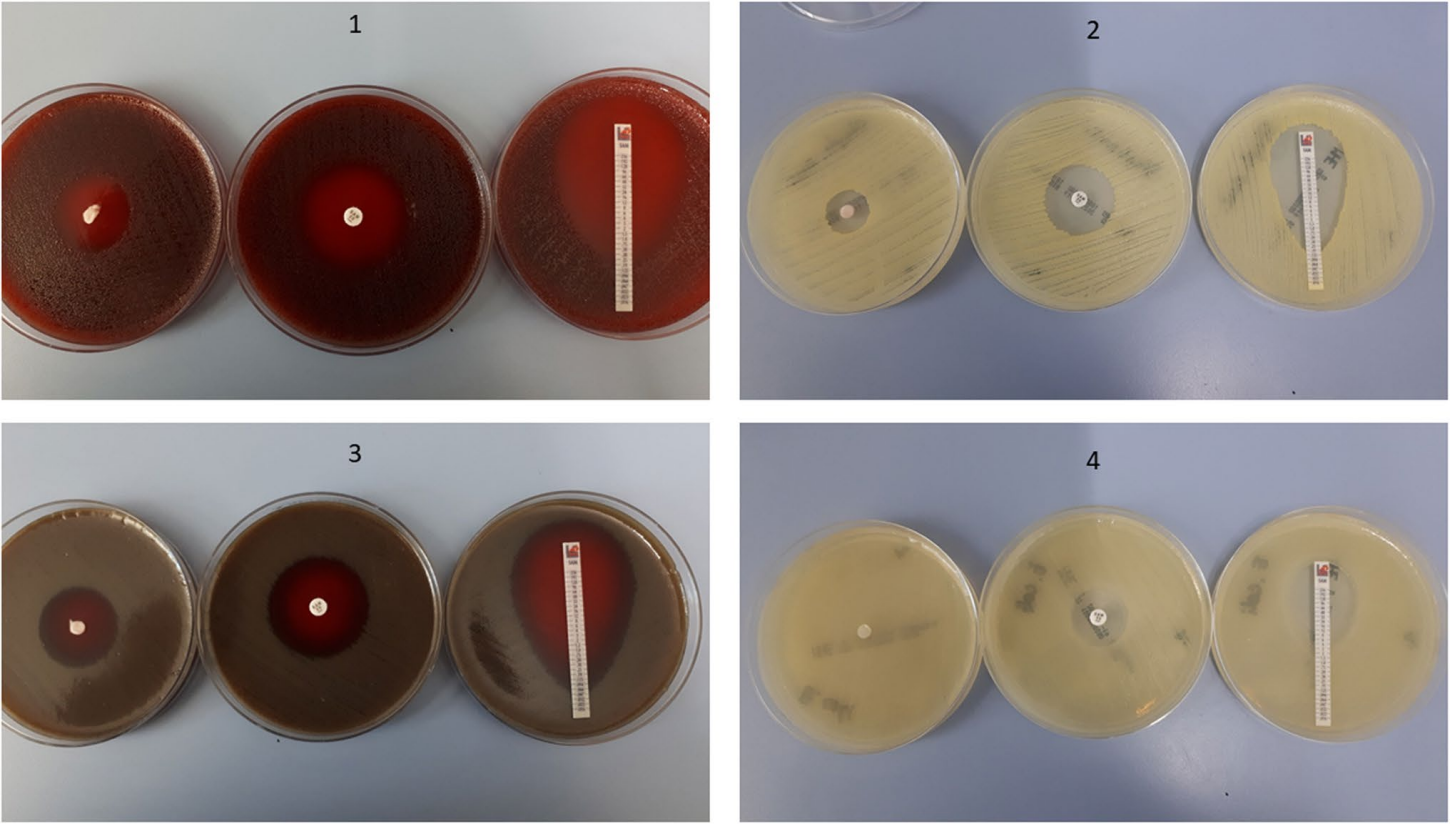
Agar diffusion test. 1: Monocultures of *Haemophilus influenzae* inoculated with a 6mm PRF disc (left), a standard ampicillin/sulbactam disc (middle), and Epsilometer test (right). 2: same as described with monocultures of *Staphylococcus aureus*. 3: same as described with monocultures of *Streptococcus pneumoniae*. 4: same as described with monocultures of *Escherichia coli*

PRF samples from 10 patients were analysed and processed for an agar diffusion test with *Escherichia coli*. No IZ was detected in seven of these 10 patients, and minor IZs could be observed in 3/10 patients. The IZ diameters for the other three patients were 8, 9, and 10mm. The average IZ diameter was thus 9mm (SD±4.37) for these samples (Table 4 and Fig. 6).

## Agar diffusion test with PRF from patients without antibiotic therapy

PRF from four patients, who did not receive any antibiotic therapy, likewise underwent agar diffusion tests with *Haemophilus influenzae, Staphylococcus aureus, Streptococcus pneumoniae*, and *Escherichia coli*. There was no IZ detected in any of the cases.

## Discussion

When ONJ is diagnosed, early surgical intervention can reduce bone and tooth loss, as well as improve quality of life for patients [26]. However, wound healing is frequently impaired in these patients, which is attributed to the poor blood supply of the soft and hard tissue in this area [6]. Because of the surgical complications, patients with ONJ (ORN or ARONJ) undergo prolonged antibiotic therapy [27]. In our hospital, patients are admitted one day prior to surgery and undergo antibiotic therapy daily from the day of admission until ten days after surgery. While hospitalized, patients undergo an intravenous regimen, which is then switched to an oral regimen on discharge, to prevent infections and re-exposed bone [6, 26, 28].

The results of this study demonstrate that the PRF membranes contain high concentrations of ampicillin/sulbactam when the antibiotics are applied to the patient intravenously before blood sampling (Fig. 5 and Table 1). This can have a lasting impact on the treatment of ONJ, particularly because it has not been fully investigated as to what extent systemically applied antibiotics reach the bony area of ONJ [29, 30]. As already stated in the introduction, it has been revealed that bacterial colonization occurs on the exposed bone even when antibiotics are administered systemically [4]. Interestingly, platelet-rich plasma (PRP), a related product, has been shown to have an antibacterial effect against individual periodontal pathogens without any previous antibiotic treatment of the patient [19]. PRP contains a high concentration of leukocytes, which release proteins such as myeloperoxidase. This, among others, can act against bacteria and fungi. Lymphocytes act through their immune system function as antigen-presenting cells, as well as producing cytokines and chemotactic signalling substances that can accompany inflammation. PRF, on the other hand, is a matrix that is formed from autologous fibrin and compressed together with platelets and leukocytes during centrifugation. In the same study as above, PRF was not found to have the same effects as PRP [19]. The respective components differ mainly in the additional calcium chloride in PRP, which could be responsible for the activation of the platelets and thus the improved antibacterial characteristics [19]. This is in line with the results of our study, in which we did not detect any antimicrobial effect of the PRF resulting from patients without prior systemic antibiosis. However, this is opposed by the discovery in a number of studies that injectable PRF (i-PRF) has an antimicrobial effect against oral *Staphylococcus aureus* and *Escherichia coli* without the addition of any antibiotic [15, 18]. In one study, the effect against *Escherichia coli* was even higher than that against *Staphylococcus aureus* [17]. It was postulated that this is caused by the release of antimicrobial proteins and by cells, most notably leukocytes, which produce cytokines in the fibrin matrix. The proportion of leukocytes in PRF decreases as centrifugation force increases (i-PRF: lower centrifugation force and high proportion of leukocytes, PRF: higher centrifugation force and thus lower proportion of leukocytes) [10]. This could explain why PRP and i-PRF have an antimicrobial effect in these previous studies, but PRF without previous ampicillin/sulbactam treatment demonstrated no such antimicrobial effect in our study [16, 17, 31].

The ability of PRF to absorb antibiotics and release them again as an antibacterial carrier has already been demonstrated in laboratory experiments. Polak et al., for example prepared PRF with the addition of metronidazole, penicillin, or clindamycin to the blood sample before centrifugation. The antimicrobial effect was tested on two bacterial strains. This antibiotic-loaded PRF showed a locally antimicrobial effect [20]. In several other studies, antibiotics were added to the blood sample either for or after centrifugation for PRF production [20, 21, 32–34]. To our best knowledge, our study reveals for the first time, that PRF is also a bio-carrier for ampicillin/sulbactam if patients previously received the antibiotic as part of the regimen to treat ONJ. This greatly enhances the described antimicrobial effect of PRF, especially for PRF products of higher centrifugation forces such as A-PRF, which we have used in our study. We proved that systemically applied antibiotics can be measured in A-PRF membranes. Furthermore, we revealed that the concentration of ampicillin in these membranes was slightly higher even than in the plasma samples. However, this effect was not statistically significant. The concentration of sulbactam in plasma and PRF was almost equal. In agar diffusion testing, we demonstrated that the PRF membranes from patients with intravenous antibiotic therapy have an antimicrobial effect on *Haemophilus influenzae, Staphylococcus aureus,* and *Streptococcus pneumoniae*. PRF clearly formed a bacterial IZ in the agar diffusion tests. The diameters of these zones and for these bacteria strains were comparable to those caused by the standard ampicillin/sulbactam discs used in sensitivity testing. We chose these bacteria as a representative selection of pathogenic bacteria present in the oropharynx, especially with regard to the fact that streptococci and *Staphylococcus aureus* cause over 64% of infected osteonecrosis cases. A further 9% are caused by bacteria such as *Haemophilus influenzae* and *Escherichia coli* [35]. In contrast to other studies, antibiotics were not added into the blood sample or the PRF product directly, but to the patient intravenously prior to blood sampling for PRF. Therefore, we demonstrated that for patients, who undergo systemic antibiotic therapy no further incorporation is necessary. On the one hand, this is a great benefit, because no further step is required, on the other hand, the antibiotic concentration within the PRF cannot be influenced and the selection of antibiotic is determined by the therapy indication. Since a calculated antibiotic therapy, should cover the expected bacteria and we further had shown that the concentrations are sufficiently high to achieve an antibacterial effect, our described method is a simple way for a local antibiotic application. The situation is different when a specific antibiotic is desired, but systemically application is not possible, for example, when the side effects exceed the positive benefit or when it is a reserve antibiotic, as with vancomycin or gentamicin. In those cases, the incorporation of patient’s blood after blood sampling could be beneficial [20, 21, 32, 33, 36, 37]. Different approaches for antibiotic instillation are described in the literature. Miron et al. described that i-PRF can be used as a drug carrier when a drug is injected into i-PRF before clot formation [33]. This is in line with Rafiee et al. who fabricated i-PRF scaffolds containing Metronidazole, Ciprofloxacin, and Minocycline. Antibiotics were added either to the blood before (group II) or after centrifugation (group I). I-PRF without the addition of antibiotics served as a control group. A sustainable antibiotic release was only shown in group I when antibiotics were added to the fabricated i-PRF before clot was formed [32, 36]. Another study by Polak et al. depicted antibacterial properties of PRF, when patients´ blood is incorporated with antibiotic drugs before the centrifugation process. [20]. Further investigation is needed, to clarify whether the incorporation of antibiotics should be performed before or after the centrifugation process.

The agar diffusion tests *with Escherichia coli* ATCC 25922 revealed that the amount of ampicillin/sulbactam released from PRF was not sufficient to inhibit the growth of this particular strain. Our measurements showed that PRF discs have a concentration of around 2-3mg/l. The MIC of *Escherichia coli* ATCC 25922 was in average 2.78mg/l as determined through our own Epsilometer tests and was within the range published by EUCAST [38]. It is also much higher than the MICs of the other three bacteria tested (0.07 – 0.19mg/l) (Table 4). This explains why the PRF did not affect the growth of *Escherichia coli* ATCC 25922, whereas an IZ was found around the technical control using the disc loaded with 10/10μg ampicillin/sulbactam as well as in the agar diffusion tests with the other three bacteria.

In spite of this observation, our *in vitro* experiments clearly have shown that ampicillin/sulbactam released from PRF inhibits the growth of surrounding bacteria. It is conceivable that this effect includes clinical *Escherichia coli* strains with a lower ampicillin/sulbactam MIC. This is also supported by data from other studies, where the antibacterial effect of PRF was greater against *Escherichia coli* than *Staphylococcus aureus* [17].

Our observation in regard of *Escherichia coli* also highlights that antibiotic resistance remains a clinically relevant problem. The proportion of resistant *Escherichia coli* against ampicillin is globally rising [38, 39]. Even though bacterial infection of ONJ is caused by *Escherichia coli* in fewer than 3% of the cases [35], ampicillin/sulbactam may be used with caution for the treatment of infected ONJ, if the proportion of *Escherichia coli* further increases in this type of infections.

In most patients, the concentration of ampicillin/sulbactam in plasma and PRF correlated, as Fig. 4 and Fig. 5 and Table 2 and Table 3 illustrate. In some patients (N=3), the plasma concentrations were very low, which correlated in one case with a lower concentration in PRF. In the other two patients, we were even unable to prepare PRF. The low concentrations may have resulted from a wrong infusion (e.g. extravasate) or a blood sampling error (e.g. saline in the peripheral venous catheter). In other patients, we detected lower concentrations in PRF than in plasma. Since the blood samples for plasma and PRF were drawn at the same time, perhaps a storage, duration, or processing error caused this phenomenon. On the other hand, as stated before, the concentration of ampicillin/sulbactam was higher than in plasma in some of the PRFs. In conclusion, however, the concentration in PRF and plasma are comparable, as Table 4 portrays.

We see the limitations of this study as being the lack of any statement regarding the effect duration and the release kinetics (how much ampicillin/sulbactam is released per defined volume). According to the results of other studies, it is possible that the antimicrobial effect lasts at least for several days [20, 21, 32]. Furthermore, we investigated the antimicrobial effects in monocultures of several bacterial strains; such conditions are not identical to the situation in the region of interest, in which bacteria grow forming a biofilm [40]. Despite the limitations of the laboratory conditions, we were still able to demonstrate that we can influence the microbial structure, respectively, reduce bacterial growth. [41, 42]. Using established laboratory methods, we were able to reveal clear evidence of the impact of our method as might take place under intraoral conditions. Another limitation is that PRF in our study was selectively performed from patients suffering from ONJ. It was not investigated whether ONJ has a positive or a negative effect to affect our findings. However, further clinical studies will be necessary to reach any decisive conclusion.

Our data reveal that PRF products are enriched in intravenously administered antibiotics and that those antibiotics are also released to the surrounding area after application. Further work will focus on release kinetics and investigate the long-term effects. The effect on microbial diversity also represents an interesting starting point for further studies. Previous studies suggest that the effect of PRF as a bio-carrier for ampicillin/sulbactam lasts for several days, which is of great clinical interest in the treatment of ONJ [20]. Depending on these results, the employment of PRF in patients with ONJ can improve the surgical outcome by reducing the frequency and severity of wound healing disorders and infections. Especially when applied to poorly perfused tissues such as in ARONJ, we see the decisive advantage offered by the local release of the enriched fibrin clot. Furthermore, PRF applied to the bone after necrosectomy can act as a lubricant layer and coverage of sharp bone edges, which affords a degree of protection to the mucosal closure.

## Data Availability

The dataset supporting the conclusions of this article is included within the article. Further clinical data and information are not publicly available because other, currently unpublished studies are based on it, but are available from the corresponding author on reasonable request.

## Abbreviations

A-PRF: advanced platelet-rich fibrin
ARONJ: antiresorptive agent-related osteonecrosis of the jaw
IZ: inhibition zone
i-PRF: injectable platelet-rich fibrin
MIC: minimum inhibitory concentration
ONJ: osteonecrosis of the jaw
ORN: osteoradionecrosis
PRF: platelet-rich fibrin
